# Beyond violence exposure: gender-specific psychological responses to violence in Ecuador – a propensity score analysis

**DOI:** 10.3389/fpsyt.2025.1716810

**Published:** 2026-01-26

**Authors:** Cintya Lanchimba, Robert Courtois, Víctor Manuel Lopez-Guerra

**Affiliations:** 1 Departamento de Economía Cuantitativa, Escuela Politécnica Nacional, Quito, Ecuador; 2 Département de Psychologie EE 1901 QualiPsy, Université de Tours, Qualité de vie et santé psychologique, Tours, France; 3 Departamento de Psicología, Universidad Técnica Particular de Loja, Loja, Ecuador

**Keywords:** alcohol, depression, Ecuador, gender differences, propensity score matching, psychological distress, violence

## Abstract

The consequences of violence differ depending on gender. This study explores the associations between exposure to violence and mental health outcomes in Ecuador, a country with high levels of violence yet limited research on its psychological effects. Using a nationally representative dataset and Propensity Score Matching (PSM) to reduce confounding bias, we examine gender-specific patterns of psychological outcomes. The analysis reveals that men exposed to violence are significantly more likely to display externalizing behaviors, including a near doubling of alcohol use (+93.6%) and heightened impulsivity, supporting theories of substance-based coping and behavioral dysregulation. In contrast, women exposed to violence show marked increases in internalizing symptoms, including depressive affect (+35.7%), perceived stress, and loneliness, reflecting emotion-focused coping strategies and affective vulnerability. While these findings support established gendered theories of violence, such as the self-medication hypothesis and emotion-focused coping, they must be interpreted with caution. The associations observed reflect robust statistical links rather than definitive causal pathways. It remains possible that pre-existing psychological vulnerabilities or unmeasured third variables contribute to both the likelihood of experiencing violence and subsequent mental health outcomes. The results underscore the importance of gender-sensitive mental health interventions: substance use and impulsivity should be targeted among men, while women’s care must be integrated with social and economic support systems. By focusing on a Latin American context, this study adds to global knowledge on the gendered psychological consequences of violence and highlights the need for longitudinal and interdisciplinary approaches.

## 1 Introduction

### 1.1 Exposure to violence

Violence, particularly physical and sexual violence, constitutes a pervasive public health issue with deep and lasting psychological consequences. According to the World Health Organization (1), violence refers to the intentional threat or use of physical force or power against oneself, another person, or a group, which results in or has a high probability of resulting in injury, death, psychological harm, maldevelopment, or deprivation. In line with WHO guidelines, violence encompasses multiple forms – physical, sexual, psychological, and economic – that may occur within intimate partnerships, families, workplaces, or wider community settings (2, 3). These violent acts may occur in private or public spaces – most notably in domestic relationships, the workplace, or the community (e.g. 4, 5).

It is important to emphasize that violence can take additional forms – such as coercive control, psychological harassment, sexual harassment, and sexual relations involving coercive manipulation – and that it occurs across multiple settings, ranging from the private sphere (couple and family relationships) to public environments (workplaces, transportation systems, and community spaces) (e.g., 4–7).

Although frequently interpreted as isolated or external events, such experiences of violence are also deeply embedded within broader social, cultural, and economic structures. They intersect not only with conditions of economic vulnerability, gender inequality, and limited institutional protection, but also with cultural norms, social hierarchies, and access to healthcare systems (see 2, 8–10). This broader framing acknowledges that while socio-economic dimensions are central, they coexist with cultural and institutional dynamics that jointly shape the prevalence and consequences of violence. This study situates its analysis primarily within the framework of intimate partner violence and gender-based violence more broadly, as these forms are particularly prevalent and clinically relevant in the Ecuadorian and Latin American context while also carrying specific implications for the emergence of psychological harm (3, 11, 12).

From a psychological standpoint, exposure to intimate partner violence (IPV) and gender-based violence more broadly may precipitate a wide array of mental health difficulties. These include, but are not limited to, anxiety disorders, major depression, post-violence stress disorder (PTSD), and substance use disorders (13–15). Framing violence primarily within the context of IPV is particularly relevant in Latin American and Ecuadorian settings, where such forms of violence are highly prevalent and clinically salient (2, 12, 16).

Conceptually, it is important to distinguish between “violence” as an exposure and “trauma” in the clinical sense. While clinical trauma is typically defined in diagnostic systems by syndrome-level criteria (e.g., PTSD) this study adopts a broader, integrative notion of psychological harm, consistent with contemporary psychological research, to capture distress-related symptoms that do not necessarily meet diagnostic thresholds (13, 17, 18). In this framework, violence is conceptualized as a potentially traumatic event, whereas psychological harm refers to the emotional, cognitive, and behavioral difficulties that may emerge or intensify following such exposure. Clarifying this distinction is essential, as the remainder of the manuscript operationalizes violence as an environmental stressor while analyzing its consequences through non-diagnostic indicators of psychological functioning (e.g., stress, depressive symptoms, impulsivity). This perspective ensures that the study captures subclinical yet clinically meaningful psychological responses, better reflecting the lived experience of affected individuals.

Operationally, in the present study, “violence” is measured as a dichotomous indicator of having ever experienced at least one form of violence (physical, psychological, sexual, or economic) within the family or intimate-partner sphere, based on nationally representative survey data from Ecuador (16). This choice reflects both the structure of the available data and the intention to capture lifetime exposure to gendered forms of violence that are particularly salient in this country.

### 1.2 Why men and women do not react in the same way: an ecological and integrative perspective

The psychological impact of violence does not occur in a vacuum but emerges from the interaction between individual, relational, community, and societal factors. Beyond the Bioecological Theory of Human Development (19), which has inspired extensive research across multiple fields (e.g., (20)), the World Health Organization’s ecological model conceptualizes violence as the result of processes operating at multiple levels – including biological vulnerabilities, family dynamics, community resources, and wider socio-cultural norms – which together shape both the risk of violence exposure and the ways in which individuals respond to it (2, 3).

Within this framework, gendered reactions to violence can be understood as the product of at least three intertwined dimensions. At the individual level, biological sex and neuropsychological functioning influence stress reactivity, emotion regulation, and susceptibility to internalizing or externalizing symptoms (17, 21, 22). At the relational level, patterns of power, economic dependence, and coercive control shape how violence is experienced and disclosed within intimate relationships (4, 23, 24). At the community and societal levels, gender norms, stigma, and institutional responses determine whether victims have access to protection, justice, and mental health care (9, 11).

Our study is anchored in an integrative conceptual framework that combines the WHO ecological model with two complementary psychological perspectives: (a) the self-medication hypothesis, which posits that individuals may use psychoactive substances to manage dysphoria and trauma-related affect (25–27), and (b) models of emotion-focused coping and rumination, which highlight how repetitive negative thinking and affective regulation strategies contribute to internalizing symptomatology, particularly among women (28).

A growing body of research suggests that these multi-level processes contribute to systematic differences in how men and women express psychological suffering. Women are more frequently exposed to patterns of intimate partner violence and economic abuse that foster emotional withdrawal, rumination, and internalizing symptomatology (28, 29). Men, in contrast, are socialized into norms of emotional restraint and self-reliance that discourage help-seeking and legitimize substance use or aggression as acceptable outlets for distress (9, 21, 29–31).

In Latin America – and particularly in Ecuador – these mechanisms are embedded in a context characterized by high levels of gender-based violence, marked social inequalities, and limited institutional protection (11, 12, 16). The ecological model is therefore especially pertinent for understanding why, in a same violent environment, men and women may follow differentiated psychological trajectories, with women more prone to internalizing responses and men to externalizing patterns. The following subsections build on this integrative perspective to describe these gendered mechanisms in more detail.

### 1.3 Internalizing psychological responses among women

Women are more likely to exhibit internalizing emotional responses following exposure to violence, including heightened levels of depressive symptoms, anxiety, and emotional dysregulation (2, 29). These psychological effects must first be understood within the broader context of women’s social positioning, which often entails greater exposure to power asymmetries, economic dependence, and social isolation (8, 32). Structural gender inequalities therefore play a central role in shaping women’s vulnerability to depressive and anxious symptomatology. Only secondarily can neurobiological processes complement this explanation, helping to clarify why some women develop persistent affective symptoms when confronted with violence exposure (17).

In Ecuador, the situation is particularly alarming: according to the National Survey on Gender-Based Violence (16), approximately 77% of Ecuadorian women have experienced at least one form of violence (psychological, physical, sexual, or economic) throughout their lives. This rate is substantially higher than the averages reported in large-scale international surveys such as the OSCE-led study on violence against women, which found that across 12 Eastern and Southern European countries, 60% of women reported psychological violence, 21% physical violence, and 7% sexual violence by an intimate partner since age 15 (33). This broader international comparison underscores the severity of the problem in the Latin American context (2). For additional comparative context, national data from France indicate similarly high levels of psychological and economic abuse (32), suggesting that gender-based violence remains pervasive even in high-income settings.

Such vulnerabilities may be particularly salient in Latin American settings, where structural and interpersonal violence are prevalent and gender inequalities remain deeply entrenched. Patterns of violence are not evenly distributed; rural and indigenous women face disproportionately high exposure, compounded by weak institutional responses, limited legal recourse, and restricted access to social protection systems (12). Despite the high prevalence of violence against women in Ecuador, documented in national statistics (16), the mental health consequences of such experiences remain largely understudied. Few studies have addressed how women emotionally process and internalize violence in culturally specific ways, and how their coping strategies may be constrained by limited access to mental health care, financial autonomy, or social support. This lack of research represents a critical gap in clinical psychology, where the understanding of culturally embedded responses is essential for designing effective, gender-sensitive interventions.

### 1.4 Externalizing psychological responses among men

The empirical literature increasingly highlights the role of gender in shaping psychological responses to violence. In the case of men, exposure to violence – particularly intimate partner violence (IPV) or other interpersonal forms – has frequently been linked to externalizing behavioral patterns, including alcohol consumption, aggression, impulsivity, and risk-taking behaviors (29, 34, 35). However, beyond the clinical and behavioral dimensions, it is crucial to situate these responses within broader sociocultural dynamics. In Latin American contexts, cultural constructions of masculinity emphasize stoicism, emotional control, and the rejection of vulnerability (9). Such expectations discourage men from expressing psychological suffering or seeking professional help, thereby reinforcing reliance on alcohol or other substances as socially “acceptable” coping strategies. This cultural reinforcement is particularly salient in Ecuador, where alcohol consumption is both widespread and normalized among men (3).

These gender role expectations deepen the dynamic, as masculinity in Latin America – and particularly in Ecuador – is socially defined through ideals of strength, emotional restraint, and self-reliance, which discourage men from acknowledging psychological suffering (9). This cultural framing positions alcohol use as a legitimate outlet for distress, while simultaneously stigmatizing the pursuit of psychological care (36). The result is a social reinforcement loop in which violence-related distress is externalized through substance use and impulsivity rather than addressed through adaptive or therapeutic pathways. From a clinical psychology perspective, this underlines the difficulty of detecting men’s psychological suffering, as it is masked by behaviors socially coded as “normal” or even expected. Neurobiological findings can enrich this understanding by illustrating how stress and violence exposure modulate reward-related circuits, thereby increasing susceptibility to maladaptive coping strategies (22, 37). Nevertheless, (21, 38) emphasizes, biological predispositions alone cannot explain men’s coping patterns; they interact continuously with sociocultural norms that dictate what is considered acceptable male behavior when responding to violence. In the Ecuadorian setting, where alcohol consumption is widespread and normalized, these intertwined cultural and biological mechanisms complicate both the recognition and the treatment of violence-related psychopathology.

### 1.5 Objectives

This study seeks to fill an important research gap by examining the gender-differentiated psychological consequences of exposure to violence in Ecuador, a country marked by one of the highest prevalence rates of gender-based violence in Latin America and significant institutional and socioeconomic disparities (12, 16). Whereas prior research has extensively documented the health and social costs of violence in Western settings, much less is known about its psychological impact in non-Western contexts, particularly in Ecuador, where structural inequalities and limited access to mental health care exacerbate vulnerability.

By adopting a clinical psychology perspective and a gender-sensitive lens, this study aims to clarify how men and women differ in their emotional responses to violence. Specifically, it focuses on the externalizing patterns often observed in men, such as alcohol consumption, and the internalizing patterns more frequent among women, such as depressive symptoms and emotional distress. This dual focus not only contributes to theory-building in gendered violence exposure responses but also provides contextually grounded insights for clinical interventions in Ecuador.

Finally, guided by the ecological and gendered frameworks outlined above, we formulate the following hypothesis:

Among individuals living in Ecuador, men exposed to violence will show higher levels of externalizing symptoms (alcohol dependence, substance use, impulsivity), whereas women exposed to violence will show higher levels of internalizing symptoms (depressive symptoms, perceived stress, loneliness), compared to non-exposed individuals of the same gender.

## 2 Methods

### 2.1 Dataset representative of the Ecuadorian population

The target population for this research comprised Ecuadorian adults spanning an age range of 18 to 80 years, encompassing both sexes. The study is based on a national mental health survey whose protocol and questionnaire were originally designed and piloted in 2019, with the aim of capturing psychological, social, and behavioral variables in the Ecuadorian adult population.

A total of 16,074 individuals were recruited via various virtual channels, including email invitations distributed after webinars organized in observance of World Mental Health Day. Additional participants were reached through public announcements by governmental and educational institutions, with the explicit support of Ecuador’s Ministry of Public Health.

Inclusion criteria were: (a) being between 18 and 80 years of age, (b) residing in Ecuador at the time of the survey, (c) being able to read Spanish, and (d) providing informed consent. Individuals who did not meet the age criteria or who declined consent were not allowed to proceed with the questionnaire. The online platform was configured with compulsory-response items for the principal study variables, so that questionnaires could not be submitted with missing responses on the instruments included in the present analyses. As a result, no cases were excluded due to item-level missing data, and the final analytical sample comprised 16,074 unique respondents. In addition, access links were individualized and monitored by the research team and clinical psychologists from the Ministry of Public Health, which prevented duplicate submissions and ensured the integrity of the dataset.

### 2.2 Procedure

Ethical approval for this study was granted in August 2021 by the Research Ethics Committee on Human Subjects at the Universidad San Francisco de Quito (CEISH code: 2021-072E). Further endorsement was obtained from the Ministry of Public Health of Ecuador. The research strictly adhered to the ethical principles outlined in the Declaration of Helsinki (39). All research instruments and the resulting database were securely maintained on an institutional server at the Universidad Técnica Particular de Loja (UTPL), configured to ensure data protection and compliance with prevailing ethical and technical standards.

Data collection was implemented between October 2021 and February 2022 through a digital platform (ArcGIS system) specifically designed for this project. Data collection in the primary care and community settings was carried out and supervised by trained clinical psychologists from the Ministry of Public Health, who were responsible for verifying the correct administration of the questionnaire and the completeness of responses. This platform hosted a meticulously curated set of questionnaires, selected based on three rigorous criteria: conciseness, demonstrated psychometric robustness, and accessibility for research purposes. Upon initial access to the platform, all participants were comprehensively informed of the study’s objectives and subsequently provided digital informed consent.

The sample is predominantly composed of young adults, with an average age of 30.91 years (SD = 10.05). Gender distribution is relatively balanced, with 8,546 men (53.17%) and 7,528 women (46.83%). Regarding marital status, the majority (54.33%) reported being single, followed by 33.13% married, 6.91% separated, 5.21% divorced, and 0.43% other. Employment status was varied: full-time employees represented 55.98% of respondents, unemployed 27.68%, part-time employees 8.61%, and informal workers 7.73%.

A key focus of the study is the impact of violence. Among the participants, 866 individuals (5.39%) reported having experienced violence, whereas 15,208 (94.61%) did not report any violence exposure. Regarding suicidal ideation, the data indicate that 15.62% of respondents have felt that life has no meaning, 3.06% have thought about committing suicide, and 0.51% have attempted suicide. The vast majority (80.81%) reported no suicidal thoughts or behaviors.

### 2.3 Measurement

This section outlines the measurement of the key constructs used in our study.

Exposure to violence: Exposure to violence was assessed through a dichotomous item in which participants reported whether they had ever experienced violence.

Sociodemographic factors: Gender was recorded as male or female. Additional sociodemographic characteristics, such as age, marital status, employment situation, subjective social class, and sleep patterns, were collected to contextualize the analyses and serve as control variables where relevant.

Psychological and behavioral consequences of violence: Mental health and behavioral outcomes were assessed with validated international instruments frequently used in clinical psychology research:

- Alcohol Use Disorder Identification Test (AUDIT): A 10-item scale developed by the World Health Organization to identify hazardous, harmful, or dependent alcohol use (25). Total scores range from 0 to 40. We used the Ecuadorian Spanish adaptation of AUDIT (40).
- Perceived Stress Scale (PSS-10): A 10-item instrument measuring the perception of stress, rated on a 5-point Likert scale, with higher scores indicating greater perceived stress (41). We employed the Spanish version whose psychometric properties have been confirmed in Ecuadorian samples (42).
- UCLA Loneliness Scale – Short version (UCLA-3): A 3-item measure assessing subjective feelings of loneliness, with scores from 0 to 9 (43).
- Acceptance and Action Questionnaire (AAQ-7): Measures psychological inflexibility and experiential avoidance; higher scores indicate greater inflexibility (44). Psychological inflexibility was assessed using the Spanish adaptation whose factorial structure and reliability have been documented in Ecuadorian and other Spanish-speaking populations (e.g., 40).
- Patient Health Questionnaire-9 (PHQ-9): A 9-item screening tool for depression assessing symptom frequency over the last two weeks (45). We used the Spanish version validated in Ecuadorian college students (46), which has shown good internal consistency and an appropriate hierarchical factor structure.
- Barratt Impulsiveness Scale (BIS-11): Evaluates impulsivity, with higher scores reflecting greater impulsiveness (47). We relied on the Spanish version, which has demonstrated adequate psychometric properties in Latin American samples (e.g., 48).

Substance use beyond alcohol: was measured using selected items from the Alcohol, Smoking and Substance Involvement Screening Test (ASSIST), which evaluates frequency of use across multiple substances (e.g. tobacco, alcohol, cannabis, cocaine, amphetamines or other stimulants, inhalants, tranquilizers or sleep medications, hallucinogens, opiates, and other substances not otherwise specified). We used the Spanish-language version following World Health Organization recommendations and previous applications in Latin American populations. Taken together, the use of these Spanish-language instruments (we used the Ecuadorian Spanish adaptation of AUDIT (40)) ensures the linguistic and cultural adequacy of the measures employed, thereby supporting the cross-cultural validity of the present findings.

### 2.4 Data processing

To strengthen causal inference in this observational study, we employed Propensity Score Matching (PSM) to estimate the association between violence exposure and psychological outcomes. This method allows us to approximate counterfactual conditions by pairing individuals exposed to violence (treatment group) with comparable individuals unexposed to violence (control group), based on observable covariates (49, 50). In our case, this is crucial given that violence is not randomly distributed across the population but shaped by sociodemographic and economic conditions. We focus specifically on estimating the Average Treatment Effect on the Treated (ATET), which captures the net psychological impact of violence among those exposed.

Although the observational design of this study precludes definitive causal inference, PSM enhances the robustness of our findings by reducing selection bias. PSM was conducted separately for men and women, using a nearest-neighbor algorithm (nn = 1). The propensity score was estimated through a probit model in which exposure to violence (yes/no) was regressed on a set of sociodemographic covariates: age, marital status, employment status, subjective social class, and sleep duration. These variables were selected *a priori* based on their documented association with both violence exposure and mental health outcomes.

To ensure adequate common support, observations with propensity scores outside the overlapping region between exposed and non-exposed participants were discarded before matching. We implemented 1:1 nearest-neighbor matching without replacement and used robust standard errors to estimate the ATET for each outcome.

Post-matching diagnostics indicated good covariate balance (standardized differences < 0.05, variance ratios ≈ 1.0), suggesting the comparability of treatment and control groups. This allows us to more reliably estimate the gender-specific psychological effects of violence (51).

## 3 Results

### 3.1 Descriptive statistics

This study examines a range of psychological, social, and behavioral factors to explore the impact of violence on mental health outcomes. **Table 1** presents the means, standard deviations, and correlation coefficients for the key variables analyzed in this study. These descriptive statistics are not only informative in themselves but also provide essential context for interpreting gendered analyses of violence exposure presented in subsequent sections.

**Table 1:** Mean and correlation matrix.

Number	Variable name	Mean	Std. Dev.	1	2	3	4	5	6	7	8	9	10	11	12	13	14	15	16	17	18	19	20
1	Age	30.9	10.05	1																			
2	Subjective social class	5.5	1.56	0.06*	1																		
3	Fear of losing job-	32.1	33.05	0.06*	−0.01	1																	
4	Sleep	6.3	2.42	0.16*	0.15*	−0.05*	1																
5	Tobacco	0.4	0.86	−0.01	0.02*	0.04*	−0.04*	1															
6	Alcohol	1.0	0.83	−0.07*	0.05*	0.05*	−0.04*	0.40*	1														
7	Cannabis	0.1	0.37	−0.09*	0.01	0.02*	−0.05*	0.22*	0.23*	1													
8	Cocaine	0.02	0.19	−0.02*	−0.003	0.01	−0.03*	0.15*	0.12*	0.40*	1												
9	Amphetamines or other stimulants	0.02	0.23	−0.02*	0.003	0.02*	−0.03*	0.09*	0.09*	0.30*	0.55*	1											
10	Inhalants	0.1	0.36	−0.05*	−0.003	0.01	−0.02*	0.08*	0.09*	0.17*	0.34*	0.30*	1										
11	Tranquilizers or sleep medications	0.2	0.58	−0.01	0.004	0.07*	−0.14*	0.11*	0.10*	0.18*	0.22*	0.26*	0.13*	1									
12	Hallucinogens	0.02	0.19	−0.04*	0.00	0.00	−0.02*	0.13*	0.09*	0.42*	0.69*	0.59*	0.37*	0.25*	1								
13	Opiates	0.01	0.18	−0.02*	0.01	0.01	−0.03*	0.12*	0.09*	0.35*	0.66*	0.57*	0.33*	0.25*	0.71*	1							
14	Other substances not otherwise specified	0.04	0.33	−0.003	0.002	0.02*	−0.04*	0.04*	0.04*	0.19*	0.32*	0.28*	0.19*	0.23*	0.36*	0.38*	1						
15	AUDIT	4.1	4.93	−0.12*	0.01	0.04*	−0.07*	0.37*	0.62*	0.22*	0.15*	0.09*	0.12*	0.11*	0.11*	0.09*	0.05*	1					
16	Perceived stress PSS	14.8	7.45	−0.31*	−0.07*	0.13*	−0.37*	0.04*	0.07*	0.11*	0.05*	0.07*	0.05*	0.23*	0.06*	0.05*	0.09*	0.15*	1				
17	Loneliness UCLA	2.9	2.49	−0.22*	−0.05*	0.09*	−0.25*	0.07*	0.12*	0.12*	0.07*	0.08*	0.05*	0.19*	0.07*	0.07*	0.10*	0.19*	0.58*	1			
18	Cognitive inflexibility AAQ	17.4	10.59	−0.29*	−0.06*	0.12*	−0.29*	0.05*	0.11*	0.13*	0.06*	0.09*	0.05*	0.24*	0.07*	0.06*	0.11*	0.19*	0.71*	0.72*	1		
19	PHQ	5.7	6.55	−0.31*	−0.06*	0.12*	−0.36*	0.08*	0.13*	0.16*	0.09*	0.11*	0.06*	0.29*	0.11*	0.10*	0.12*	0.20*	0.67*	0.67*	0.78*	1	
20	BIS	62.3	9.93	−0.25*	−0.06*	0.10*	−0.28*	0.11*	0.14*	0.16*	0.09*	0.08*	0.08*	0.17*	0.09*	0.07*	0.09*	0.22*	0.58*	0.47*	0.55*	0.57*	1

*p<0.05.

The sample was composed of 16,073 adults with a relatively young profile (M = 30.91, SD = 10.05) and a balanced gender distribution. Most respondents located themselves in the middle socioeconomic tier (M = 5.46, SD = 1.56). These baseline indicators highlight the diversity of demographic backgrounds in the dataset and provide a foundation for examining psychological outcomes related to violence.

Substance use behaviors, assessed using the Alcohol, Smoking, and Substance Involvement Screening Test (ASSIST), revealed consistent patterns of comorbidity: individuals reporting one form of substance use were significantly more likely to report others. Alcohol consumption, assessed by the AUDIT, emerged as the most prevalent and showed positive associations with multiple substance categories, confirming its central role in maladaptive coping strategies within the Ecuadorian context. In terms of psychological functioning, mean scores on the Perceived Stress Scale (PSS = 14.79, SD = 7.45), the UCLA Loneliness Scale (M = 2.91, SD = 2.49), and the Patient Health Questionnaire (PHQ-9 = 5.68, SD = 6.55) indicated moderate but clinically relevant levels of stress, loneliness, and depressive symptoms. Cognitive inflexibility, captured by the AAQ (M = 17.43, SD = 10.59), further highlighted the prevalence of maladaptive coping among participants.

Correlation analyses (**Table 1**) confirmed strong associations between substance use and psychological distress, with alcohol use in particular showing consistent links to stress, loneliness, and depressive symptoms. Age negatively correlated with these mental health indicators, underscoring the vulnerability of younger individuals to adverse psychological outcomes when exposed to violence.

These descriptive statistics highlight the psychological vulnerabilities prevalent in the Ecuadorian population, particularly among younger individuals and those involved in substance use, and lay the groundwork for the gender-disaggregated Propensity Score Matching (PSM) analyses presented in the following subsections (3.1 for men, 3.2 for women, and 3.3 for comparative interpretation). Unless otherwise specified, the percentages reported in parentheses for PSM results refer to the relative difference between individuals exposed to and unexposed to violence in the matched sample, calculated as the ATET divided by the mean outcome among matched controls multiplied by 100.

### 3.2 Psychological impact of violence among men

The psychological consequences of violence are particularly pronounced among men, with consistent increases across substance use, alcohol consumption, psychological distress, and impulsivity. Men who have been exposed to violence show systematically higher likelihoods of engaging in externalizing behaviors, including alcohol misuse and illicit drug use, compared to non-exposed men.

The most striking difference is observed in alcohol use (AUDIT), which increases by 3.81 points (a relative increase of 93.6% with respect to matched controls), compared to a 41.1% increase among women.

Men also exhibit higher levels of psychological distress. Depressive symptoms increase by 5.62 points (45% relative increase), perceived stress (PSS) rises by 4.59 points (31% relative increase), and cognitive inflexibility (AAQ) increases by 8.51 points (48.8% relative increase), indicating marked emotional dysregulation and reduced adaptive flexibility.

In terms of substance use, tobacco consumption increases by 37%, while alcohol use rises by 33.3%. Men exposed to violence are also significantly more likely to use illicit drugs compared to women, with cannabis use increasing by 26.3% and amphetamine use by 17.2%. Additional increases are also observed for cocaine, inhalants, tranquilizers, hallucinogens, and opiates, reinforcing the pattern of polysubstance use among men exposed to violence.

In addition, impulsivity (BIS) increases by 6.15 points (41% relative increase), compared to 4.13 points (29.5% relative increase) in women, reinforcing a greater likelihood of externalizing behaviors among men.

### 3.3 Psychological impact of violence among women

**Table 2** presents the estimated Average Treatment Effect on the Treated (ATET) for various psychological outcomes, separately for women and men. Among women who have experienced violence, significant differences are observed in substance use, perceived stress, depressive symptoms, and impulsivity.

**Table 2:** Psychological outcomes among women exposed to violence (PSM estimates).

Outcome variable	Women
tobacco	0.208 (SE = 0.042) [0.125, 0.291]
alcohol	0.225 (SE = 0.045) [0.136, 0.313]
cannabis	0.086 (SE = 0.023) [0.040, 0.131]
cocaine	0.014 (SE = 0.009) [–0.003, 0.032]
amphetamines or other stimulants	0.058 (SE = 0.018) [0.023, 0.093]
inhalants	0.025 (SE = 0.018) [–0.010, 0.061]
tranquilizers or sleep medications	0.225 (SE = 0.041) [0.145, 0.305]
Hallucinogens	0.038 (SE = 0.012) [0.014, 0.062]
opiates	0.010 (SE = 0.008) [–0.005, 0.025]
other substances	0.109 (SE = 0.028) [0.054, 0.165]
AUDIT	1.737 (SE = 0.244) [1.258, 2.215]
PSS	4.240 (SE = 0.350) [3.554, 4.926]
UCLA	1.313 (SE = 0.129) [1.060, 1.566]
AAQ	6.562 (SE = 0.557) [5.470, 7.655]
PHQ	3.682 (SE = 0.364) [2.968, 4.395]
BIS	4.127 (SE = 0.469) [3.207, 5.047]

The first column lists the psychological and behavioral outcome variables analyzed in the Propensity Score Matching (PSM) models. The second column reports the Average Treatment Effect on the Treated (ATET) for women exposed to violence compared to non-exposed counterparts. Estimates are presented as effect sizes, with their standard errors (SE) and 95% confidence intervals in brackets.

Women who have been exposed to violence show a substantial increase in psychological distress compared to their non-exposed counterparts. Specifically, perceived stress (PSS) increases by 4.24 points, representing a 28.7% rise relative to the control group. Similarly, depressive symptoms (PHQ) increase by 3.68 points, translating to a 35.7% increase in depressive symptomatology. In addition to stress and depression, women exposed to violence also report higher levels of loneliness (UCLA), with an increase of 1.31 points, or 45%. Similarly, cognitive inflexibility (AAQ), a psychological marker associated with maladaptive stress responses, increases by 6.56 points, or 37.7%, which may indicate difficulties in adapting to violent experiences and regulating emotional responses.

Substance use patterns among women also change after exposure to violence. Alcohol consumption increases by 22.5%, while tobacco use increases by 20.8%. Additionally, the use of tranquilizers rises by 22.5%, which may indicate self-medication as a coping strategy. However, the use of illicit drugs such as cannabis, cocaine, and amphetamines remain relatively low, with changes ranging between 1.4% and 10.9%., indicating that while some women resort to illicit substances, the most common coping mechanisms remain alcohol and prescription sedatives.

Impulsivity and risk-taking behavior, as measured by the BIS scale, also show a 4.13-point increase (29.5% relative increase) among women exposed to violence.

**Figure 1:**
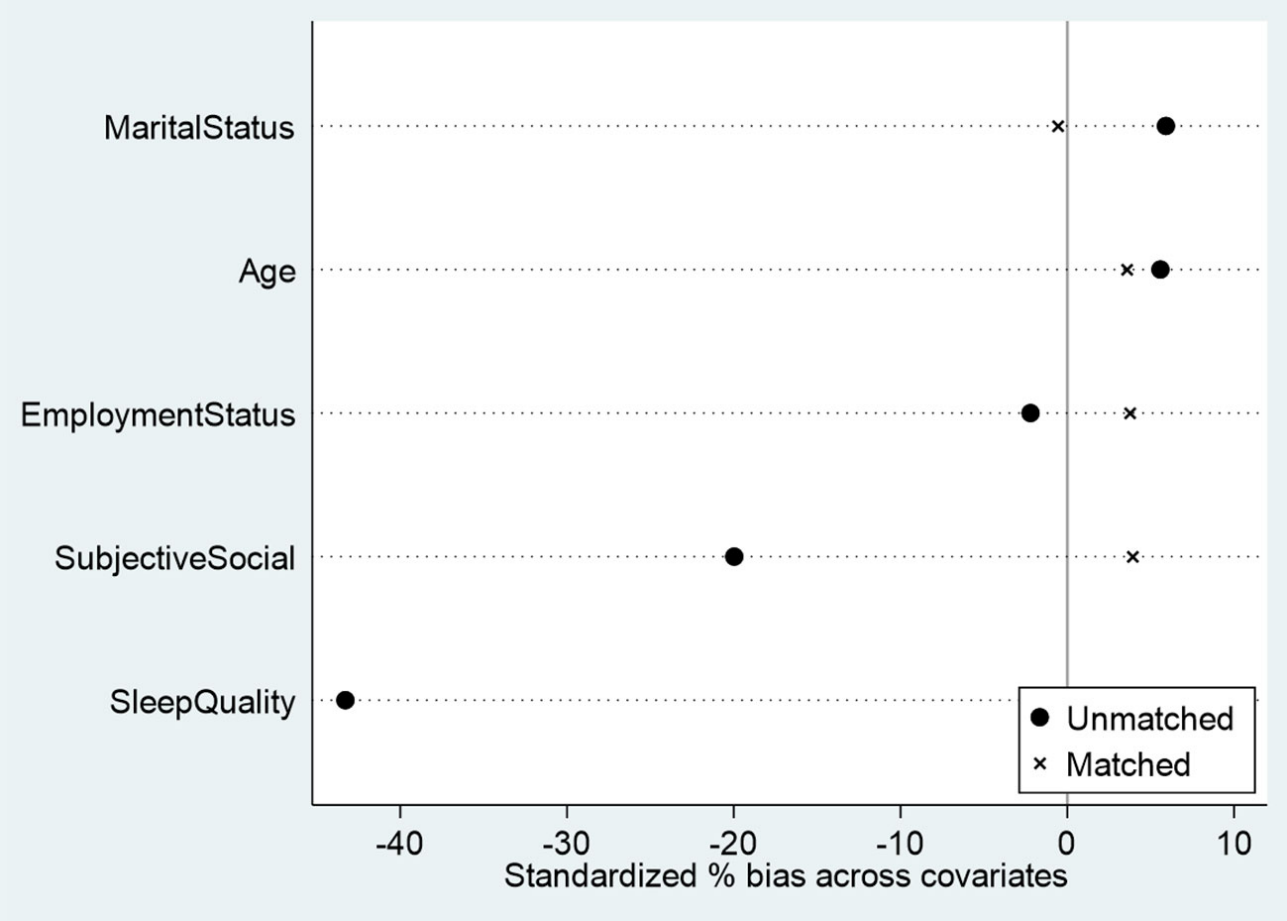
Covariate balance before and after propensity score matching (men).

**Figure 2:**
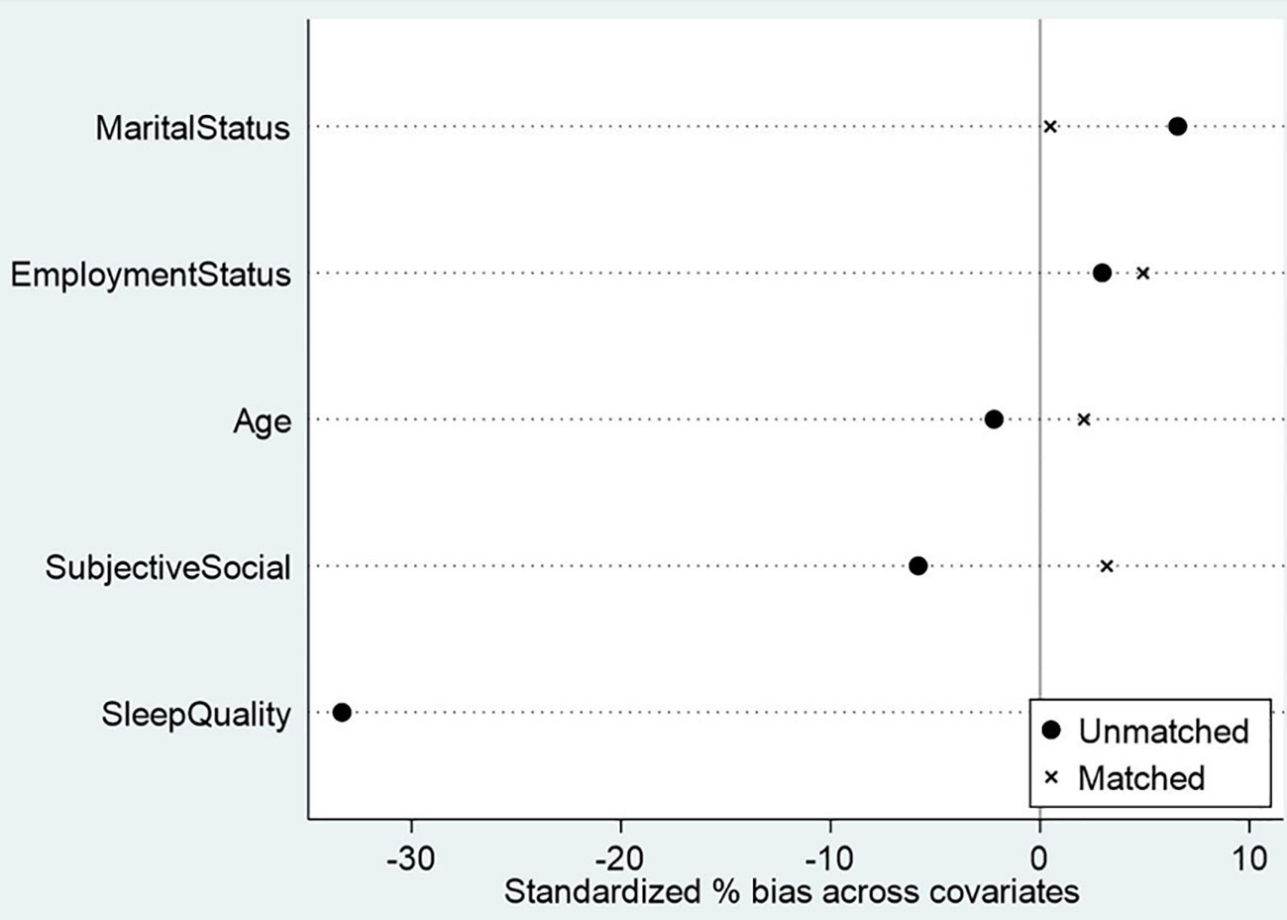
Covariate balance before and after propensity score matching (women).

Before estimating the psychological effects of violence, we evaluated the quality of the propensity score matching procedure separately for men and women (**Figures 1**, **2**). **Figures 1**, **2** present the covariate balance (love plots) before and after matching. In both groups, several covariates displayed substantial standardized mean differences prior to matching- particularly subjective social status, sleep quality, and employment status. After matching, however, the standardized biases for all covariates were reduced to below 5%, indicating excellent balance and confirming that the matched samples are highly comparable. This ensures that the estimated treatment effects reported in **Tables 2**, **3** are unlikely to be driven by baseline differences and can therefore be interpreted as robust estimates of the psychological impact of violence.

**Table 3:** Psychological outcomes among men exposed to violence (PSM estimates).

Outcome variable	Men
tobacco	0.370 (SE = 0.114) [0.147, 0.592]
alcohol	0.333 (SE = 0.096) [0.146, 0.521]
cannabis	0.263 (SE = 0.058) [0.150, 0.376]
cocaine	0.172 (SE = 0.050) [0.075, 0.269]
amphetamines or other stimulants	0.172 (SE = 0.048) [0.078, 0.266]
inhalants	0.208 (SE = 0.053) [0.103, 0.312]
tranquilizers or sleep medications	0.376 (SE = 0.067) [0.245, 0.507]
Hallucinogens	0.189 (SE = 0.049) [0.093, 0.285]
opiates	0.136 (SE = 0.047) [0.044, 0.228]
other substances	0.167 (SE = 0.052) [0.064, 0.269]
AUDIT	3.807 (SE = 0.666) [2.502, 5.112]
PSS	4.599 (SE = 0.592) [3.439, 5.759]
UCLA	2.009 (SE = 0.231) [1.556, 2.461]
AAQ	8.513 (SE = 0.934) [6.682, 10.345]
PHQ	5.617 (SE = 0.606) [4.429, 6.804]
BIS	6.154 (SE = 0.807) [4.572, 7.736]

The first column lists the psychological and behavioral outcome variables analyzed in the Propensity Score Matching (PSM) models. The second column reports the Average Treatment Effect on the Treated (ATET) for men exposed to violence compared to non-exposed counterparts. Estimates are presented as effect sizes, with their standard errors (SE) and 95% confidence intervals in brackets.

## 4 Discussion

### 4.1 Summary of main findings

This study demonstrates that exposure to violence is associated with profound psychological consequences, but these consequences manifest differently according to gender.

For men, the results indicate a pattern of externalization, characterized by higher levels of alcohol use, illicit substance use, and impulsivity. These outcomes are consistent with the self-medication hypothesis, which posits that individuals may rely on substances as a maladaptive coping strategy following violence exposure (27, 30). Our findings extend this literature by demonstrating that, even after balancing covariates with PSM, violence-exposed men in Ecuador present a clear externalizing profile, particularly through alcohol misuse, an element strongly embedded in regional masculine norm.

For women, in contrast, the findings point to a pattern of internalization, marked by elevated symptoms of depression, heightened stress, loneliness, and cognitive rigidity. These results align with research indicating that women exposed to intimate partner violence are at heightened risk of developing affective disorders and social withdrawal (13, 18). Consistent with emotion-focused coping models, our results suggest that women’s affective vulnerability and social disconnection function as key pathways through which violence affects mental health.

Taken together, the psychological sequelae of violence are multidimensional, involving both behavioral dysregulation and emotional suffering, and gender-specific pathways mediate these outcomes. This divergence between externalizing and internalizing profiles underscores the importance of considering gender not merely as a control variable but as a central determinant in shaping the psychological impact of violence.

### 4.2 Interpretation and theoretical integration

These results reinforce and extend several frameworks in clinical and social psychology. First, the pattern observed among men is consistent with the *self-medication hypothesis*, which posits that individuals use psychoactive substances to regulate post-traumatic affect and dysphoria (26, 27). In our data, men exposed to violence display a markedly higher propensity toward alcohol problems (as indexed by AUDIT; 25) and greater impulsivity (47). Crucially, in the Ecuadorian and broader Andean context, alcohol consumption is socially normalized among men and often embedded in masculine identity rituals (e.g., party, community labor mingas). This cultural acceptance likely amplifies the externalizing trajectory observed in our male sample.

Second, the gendered divergence between externalizing (men) and internalizing (women) profiles aligns with stress-coping models and evidence on violence responses. Women exposed to violence show elevated depressive symptoms and perceived stress in numerous cohorts, and ruminative styles exacerbate these outcomes. The heightened loneliness and cognitive rigidity observed in Ecuadorian women may also reflect local structural barriers, including economic dependency and limited access to formal help-seeking services.

Third, our interpretation bridges clinical mechanisms with structural determinants. Women’s mental-health burden is frequently compounded by coercive control, economic abuse, and constrained help-seeking, which intensify and prolong distress (4, 8). In Ecuador and the Andean region, high prevalence of intimate partner violence, combined with persistent gendered norms around caretaking and silence, may intensify internalizing symptoms. Consistent with this literature, our female subsample shows salient elevations in depressive affect, stress, and loneliness-markers of internalizing distress and social disconnection.

For men, the pattern fits with scholarship on gendered socialization: norms valorizing emotional restraint and self-reliance can inhibit disclosure and professional help-seeking. This intersects with Latin American constructs such as *machismo*, which encourage endurance and discourage emotional expression.

Thus, a clinically coherent picture emerges men’s outcomes map onto an externalizing pathway shaped by socio-cultural norms around alcohol and emotional suppression, whereas women’s outcomes reflect an internalizing pathway influenced by structural constraints, affective vulnerability, and social disconnection.

### 4.3 Practical implications

The gender-differentiated outcomes observed have important implications for mental health policy and clinical intervention design.

For men, interventions should prioritize substance use treatment. Programs integrating Cognitive-Behavioral Therapy for addiction, trauma-informed motivational interviewing, and culturally adapted peer-support strategies may be particularly effective in Ecuador, where alcohol-based socialization is ubiquitous. Public health messaging should challenge harmful masculine norms that stigmatize emotional vulnerability.

For women, interventions should adopt a multilevel perspective. Programs must simultaneously address structural inequalities (economic dependency, coercive control), promote social reconnection, and provide gender-sensitive trauma therapy. Community-based interventions may mitigate isolation and promote supportive networks.

In both cases, violence-informed and gender-responsive care is essential, integrating cultural, structural, and psychological determinants of distress.

### 4.4 Limitations

Several limitations of this study warrant consideration. First, the cross-sectional design precludes strong causal inference. Although PSM reduces observed confounding and substantially improved covariate balance (see **Figures 1**, **2**), the study remains observational, and unmeasured confounding cannot be excluded.

Second, reliance on self-report may introduce bias, particularly for substance use among men. Third, regional cultural heterogeneity in Latin America may limit generalizability. Future studies should incorporate longitudinal designs and culturally grounded qualitative work to enrich understanding of gendered violence responses.

### 4.5 Future directions

Future research should examine temporal dynamics through longitudinal designs. Qualitative and mixed-methods approaches can illuminate how men and women narrate violence, cope with distress, and engage with help-seeking within specific cultural frameworks. Interdisciplinary collaboration will be essential for developing integrated interventions. Intersectional factors-ethnicity, rurality, sexual orientation, disability-should also be incorporated to refine understanding of violence consequences in Ecuador and the Andean region.

## 5 Conclusion

This study shows that violence has profound psychological consequences that manifest differently by gender. Men tend to externalize distress through alcohol consumption, substance use, and impulsivity, whereas women are more prone to internalizing responses such as depression, stress, and loneliness. These gender-specific pathways highlight how structural, cultural, and psychological processes jointly shape responses to violence.

Addressing these differentiated outcomes requires violence-informed and gender-sensitive, culturally informed, and violence-informed interventions that integrate clinical support with broader strategies to reduce economic dependency, social isolation, and harmful gender norms.

## Data Availability

The raw data supporting the conclusions of this article will be made available by the authors, without undue reservation.
